# Mental health of new undergraduate students before and after COVID-19 in China

**DOI:** 10.1038/s41598-021-98140-3

**Published:** 2021-09-22

**Authors:** Peng Lu, Lei Yang, Chongjian Wang, Guoxin Xia, Hao Xiang, Gongbo Chen, Ning Jiang, Tingting Ye, Yucheng Pang, Hongwei Sun, Lailai Yan, Zhenguo Su, Jane Heyworth, Rachel Huxley, Jane Fisher, Shanshan Li, Yuming Guo

**Affiliations:** 1 School of Public Health and Management, Binzhou Medical University, Yantai, Shandong China; 2 Department of Epidemiology and Statistics, School of Public Health, Hebei Medical University, Hebei Key Laboratory of Environment and Human Health, Shijiazhuang, China; 3 School of Public Health, Zhengzhou University, Zhengzhou, Henan China; 4 The Second School of Clinical Medicine, Binzhou Medical University, Yantai, Shandong China; 5 Department of Global Health, School of Health Sciences, Wuhan University, 115 Donghu Road, Wuhan, Hubei China; 6 Department of Epidemiology and Preventive Medicine, School of Public Health and Preventive Medicine, Monash University, Melbourne, VIC Australia; 7 Department of Human Resources, Binzhou Medical University, Yantai, Shandong China; 8 School of Public Health, Peking University, Beijing, China; 9 School of Population and Global Health, The University of Western Australia, Perth, WA Australia; 10 Deakin University, Melbourne, VIC Australia

**Keywords:** Psychology, Environmental social sciences

## Abstract

The purpose of this study was to examine the changes in severity of anxiety and depression symptoms, stress and sleeping quality after three months of mass quarantine for COVID-19 among undergraduate fresh students compared to their pre-COVID-19 measures. We used participants from the Chinese Undergraduate Cohort (CUC), a national prospective longitudinal study to examine the changes in anxiety and depression symptoms severity, stress and sleep quality after being under mass quarantine for three months. Wilcoxon matched pair signed-rank test was used to compare the lifestyle indicators. Severity of anxiety, depression symptoms, stress and sleep quality were compared with Wilcoxon signed-rank test. We used generalized estimating equation (GEE) to further quantify the change in mental health indicators and sleep quality after the COVID-19 mass quarantine compared to baseline. This study found that there was no deterioration in mental health status among Chinese new undergraduate students in 2020 after COVID-19 mass quarantine compared with the baseline measures in 2019. There was an improvement in sleep quality and anxiety symptoms. After adjusting for age, sex, exercise habit, time spent on mobile gadgets, and time spent outdoors, year 2020 was significantly associated with severity of depression symptoms in males (OR:1.52. 95%CI:1.05–2.20, *p*-value = 0.027). Year 2020 was significantly associated with the improvement of sleeping quality in total (OR:0.45, 95%CI:0.38–0.52, *p* < 0.001) and in all the subgroups. This longitudinal study found no deterioration in mental health status among Chinese new undergraduate students after three months of mass quarantine for COVID-19.

## Introduction

The unprecedented outbreak of COVID-19 influenced people’s lives in every aspect. On 30 January 2020, the World Health Organization declared the disease a Public Health Emergency of International Concern^1^. Public panic and mental distress reactions to the fear of becoming infected by this unknown virus have been reported^2,3^. Further, the responses to the COVID-19 outbreak are expected to lead to mental health impacts^2^. Mental health refers to cognitive, behavioral, and emotional well-being. It can affect physical health. After the outbreak, many countries implemented mass quarantine (lockdown) to contain the spread of disease, which restricted people from usual outdoor activities (e.g., exercise or social). In turn these restrictions might result in loneliness, anxiety, depression and sleeping disorders. College students, as an outdoor active group^4^, might be affected mentally by this restriction. Rubin et al. indicated that widespread lockdown would inevitably have a psychological effect due to the sense of being trapped and the belief that authorities are not acting for the benefits of those within the lockdown cities^4^.

Several cross-sectional surveys have been performed to evaluate the mental impact of COVID-19 outbreak. In a Chinese nationwide cross-sectional study of 52,730 citizens surveyed between 31 January 2020 and 10 February 2020, individuals aged 18–30 years old and individuals > 60 years old demonstrated higher psychological distress scores than other age groups^3^. All the cities in mainland China started to implement mass quarantine from 23–25 January 2020. Another cross-sectional study among Chinese adults demonstrated that the high prevalence of mental health problems during the COVID-19 outbreak was positively associated with social media exposure^5^. A third cross-sectional study recruited 1,912 Chinese university students online and collected data between 20 March and 10 April 2020. The result found prevalent psychiatric symptoms, with 46.55% college students showing depressive symptoms, and 34.73% students reporting anxiety symptoms^6^. These data suggest that young adults are a vulnerable group for the potential mental health impacts of the response to COVID-19 outbreak. However, these existing studies were limited by the cross-sectional design, which did not measure mental health status before the COVID-19 outbreak. This makes them unable to evaluate psychological changes after COVID-19 compared to baseline status.

To fill the knowledge gap, we used the fresh students from Chinese Undergraduate Cohort study (CUC) to examine the changes in severity of anxiety and depression symptoms, stress and sleeping quality after three months of mass quarantine for COVID-19 among undergraduate students compared to their pre-COVID-19 measures. We also examined whether the changes in mental health status varied by sex, family income groups, and local outbreak level of COVID-19.

## Methods

### Study design and participants

In 2019, the CUC, a prospective longitudinal study, was founded by four universities located in four provinces (Shandong, Hebei, Henan and Hubei), China, in 2019. The original purpose of the CUC was to investigate the effects of lifetime environmental exposures (e.g., indoor air pollution, outdoor air pollution, residential green space, internal metal exposure, and nutrition) and behaviors (smoking, drinking, physical activity) on health outcomes (physical and mental health). We used random cluster sampling method to choose study subjects. Students enrolled in the cohort universities in 2019 were chosen as a cluster. Those who finished both questionnaires were included. The other cases were excluded. We recruited 5,181 students who joined the universities between 23–29 August 2019. Written informed consent was obtained from those students before participation. The CUC was approved by Binzhou Medical College ethics committee. The entire study was approved by Binzhou Medical College ethics committee.

### Procedures

The baseline data collection was conducted during 16 September 2019 and 23 October 2019, at the time of the compulsory college-entry health examinations. This included a self-reported questionnaire, lung-function test, and blood sample collection. The content of the questionnaire included demographic data (age, sex, birthday, home address, height, weight); lifestyle data (smoking, drinking, physical activity, length of time spent outdoors, length of time spent on their mobile gadgets) and mental health data (symptoms of anxiety, depression, self-reported stress and sleep quality). The follow-up data collection was conducted during 16 April 2020 and 23 April 2020, almost three months after the implement of mass quarantine. No incentive measures were used to increase the response rate. We distributed the questionnaire through class committee. Those who took part in both the first and the follow-up study were chosen as our study subjects.

#### Outcomes for mental health and sleep quality

We used a 7-item generalized anxiety disorder (GAD-7) scale (range 0–21) to assess the severity of anxiety disorders. Scores can be interpreted as normal (0–4), mild (5–9), moderate (10–14), and severe (15–21) anxiety^7^. In this study, 10 points was set as a cut-off score as clinically significant symptoms of anxiety^8^. Individuals with scores ≥ 10 points were categorized into anxiety group. The 9-item, patient health questionnaire depression (PHQ-9) scale (range 0–27) was used to assess the severity of depressive disorders. Scores can be interpreted as normal (0–4), mild (5–9), moderate (10–14), and severe (15–27) depressive disorders^9^. Individuals with scores ≥ 10 points were categorized into depression group^10^. Self-reported stress was categorized into five groups, that is no, a little, mild, moderate, and severe. No stress and a little stress were the reference group. Mild, moderate and severe stress group were categorized as stress group. The self-reported stress scale is not a validated measure. Pittsburgh sleep quality index (PSQI) scale (range 0–21) was used to assess sleep quality. In this study, 5 points was set as a cut-off score to distinguish between normal sleep quality (0–5) and poor sleep quality (> 5). Bachaus et al. indicated that PSQI global score > 5 has a good sensitivity and specificity as a marker for sleep disturbances^11^.

To measure change in mental health status of individuals, 2020 scores were subtracted by 2019 scores for each of the measures used in the study. Then we categorized the results into improved, no change and worsened.

#### Severity of 2019-nCoV pandemic

The severity of 2019-nCoV pandemic was defined based upon city-level COVID-19 confirmed cases per million inhabitants by 5 March 2020. Because there is no universal definition about low/high pandemic areas, we separated the study subjects into almost even two parts. That is to say we used 50% quantile of the cases per million per city as cut off. Our flexible approach during the COVID-19 pandemic-cities with more than 6 cases per million population was categorized as high COVID-19 pandemic areas.

#### Lifestyle factors

The following lifestyle behaviors were dichotomized and the definitions used were: smoking- at least one cigarette per day for half a year; drinking-consumption of alcohol at least once per month for the past three months; and physical activity at least once per week for the past three months. The following lifestyle behaviors were continuous and the definitions used were: time spent outdoors-daily average time spent outdoors for the past three months; time spent on their mobile gadgets-daily average time spent watching mobile phones or tablets for the past three months. According to previous studies, the median times per day outdoors during weekdays were 1.04 h^12^. Therefore, we dichotomized daily time spent outdoors into ≤ 1 h group and > 1 h group. We also dichotomized daily average time spent on mobile phones by 3 h, as previous longitudinal studies suggested^13,14^. We categorized the home address of the study subjects into urban and rural areas. The urban and rural information was collected from China’s 2010 population census^15^. The annual family income was categorized in to low- and high-income groups according to the cutoff points of 50,000RMB (≈US$7,580) as previous study suggested^16^.

To examine the impacts of COVID-19 on mental health and sleep quality, we re-surveyed the cohort participants during 16–27 April 2020 after lifting mass quarantine (23–25 January 2020) in China.

### Statistical analysis

We used Wilcoxon matched pair signed-rank test to compare the lifestyle indicators. Severity of anxiety, depression symptoms, stress and sleep quality were compared as grading variables with Wilcoxon signed-rank test.

We used generalized estimating equation (GEE) to further quantify the change in mental health indicators and sleep quality after the COVID-19 mass quarantine compared to baseline. GEE is an extension of generalized linear models (GLM) using correlation matrix to adjust for correlations between observations^17^. It is often applied for longitudinal study with repeated measurements^18^. The dependent variables were depression (PHQ-9 score ≥ 10), anxiety (GAD-7 score ≥ 10), stress (self-reported mild, moderate and severe stress), good sleeping quality (PSQI score ≤ 5). Covariates included age, sex, exercise habit, time spent outdoors, and time spent on mobile gadgets. We used two models to check the associations. Model 1 adjusted for age and sex. Model 2 adjusted for age, sex, exercise habit, time spent outdoors and time spent on mobile gadgets. We used different models by adding different covariates to control the confounding effects of life-style factors such as exercise, outdoor time, mobile-occupying time. Intra-group comparison was realized by adding interaction term between year and group.

Sensitivity analyses were performed to check the robustness of our findings. For example, we used different cut-offs (5 points) for anxiety and depression to check whether the association with year 2020 changed or not. We also used different cut-offs (around 75% quantile of the cases per million-15 cases per million) for COVID-19 risk areas to check if there any change in the main results.

All statistical analyses were performed in R software (version 3.6.1). The “geeglm” package was used to perform the generalized estimating equations model and the “wilcox” package was used to perform signed-rank test. All methods were carried out in accordance with relevant guidelines and regulations.

## Results

Table 1 shows the distribution of demographic characteristics, lifestyle characteristics, mental health status, and sleep quality in 2019 and 2020 (after lifting lockdown). There were in total 5,181 participants from 307 cities in China. The distribution of the study participants is shown in supplementary materials (Figure S1). Among them 2,410 individuals were living in high COVID-19 pandemic areas. We found that there was no significant difference in the smoking, drinking and physical activity habits between the 2019 baseline and the 2020 follow up. The time spent outdoors changed significantly from 13% students staying outside ≤ 1 h in 2019 to 60% in 2020. Time spent on mobile gadgets increased significantly from 40% individuals spending more than 3 h per day in 2019 to 71% in 2020. There were no changes in self-reported stress or depression. The changing anxiety scores were found in the normal and mild anxiety symptom groups. The proportion of students with mild anxiety symptoms decreased and those who were normal increased (Table S1). The proportion of good sleep quality increased from 80% in 2019 to 89% in 2020.

**Table 1:** The distribution of demographic characteristics, lifestyle characteristics, mental health status and sleep quality between 2019 and lockdown.

		Distribution		p-value
		2019 N (%)	2020 N (%)	
**Demographic characteristics**				
Age	14–17	418 (8.1%)	28 (0.5%)	
	18–24	4,763 (91.9%)	5,153 (99.5%)	
Sex	Female	3,220 (62.2%)	3,220 (62.2%)	
	Male	1,961 (37.8%)	1,961 (37.8%)	
Major	Medicine	5,012 (96.7%)	5,012 (96.7%)	
	Other	1,69 (3.3%)	1,69 (3.3%)	
Province	Hubei	72 (1.4%)	72 (1.4%)	
	Other	5,109 (98.6%)	5,109 (98.6%)	
Socioeconomic	Urban	2,600 (50.2%)	2,600 (50.2%)	
	Rural	2,581 (49.8%)	2,581 (49.8%)	
	≤ 6 cases per million	-	2,771 (53.5%)	
2019-nCoV pandemics	> 6 cases per million	-	2,410 (46.5%)	
Average annual family income (RMB)	< 50,000	2,612 (50.4%)	-	
	≥ 50,000	2,569 (49.6%)	-	
**Lifestyle characteristics**				
Smoke				0.40
	No or occasional	5,147 (99.3%)	5,142 (99.2%)	
	Yes	34 (0.7%)	39 (0.8%)	
Drink alcohol				0.95
	No or occasional	5,002 (96.5%)	4,996 (96.4%)	
	Yes	179 (3.5%)	185 (3.6%)	
Exercise				0.49
	No	1,395 (26.9%)	1,438 (27.8%)	
	Yes	3,786 (73.1%)	3,610 (72.2%)	
Time spent outdoors				< 0.001
	≤ 1 h	696 (13.4%)	3,146 (60.7%)	
	> 1 h	4,485 (86.6%)	2,035 (39.3%)	
Time spent on mobile gadgets				< 0.001
	≤ 3 h	3,117 (60.2%)	1,484 (28.6%)	
	> 3 h	2,064 (39.8%)	3,697 (71.4%)	
**Mental health status**				
Anxiety				0.007
	No	5,001 (96.5%)	4,991 (96.4%)	
	Yes	180 (3.5%)	190 (3.7%)	
Depression				0.881
	No	4,885 (94.3%)	4,805 (92.7%)	
	Yes	296 (5.8%)	376 (7.2%)	
Stress				0.202
	No	3,643 (70.3%)	3,520 (68.0%)	
	Yes	1,538 (29.7%)	1,657 (32.1%)	
**Sleep quality**				
Sleep quality				< 0.001
	Good	4,150 (80.1%)	4,614 (89.1%)	
	Poor	1,019 (19.7%)	555 (10.7%)	

**Figure 1:**
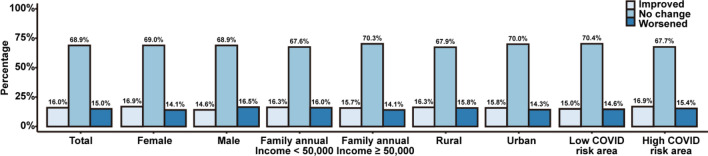
The change of severity of depression symptoms by sex, family income, urban/rural areas, and COVID-19 risk areas between 2019 and 2020. Notes: Statistically significant difference was found in male (*p*-value = 0.016).

Figure 1 shows the change in depression symptoms by sex, family income, and COVID-19 risk areas between 2019 and 2020. For each around 70% of the participants’ depression symptoms remain unchanged, and for around 15% they either worsened or improved. In the subgroup analysis, there were more individuals with improved depression symptoms than those with worsen symptoms, except the male group. There were 14.6% males showing improved depression symptoms, 16.5% with worsen symptoms.

**Figure 2:**
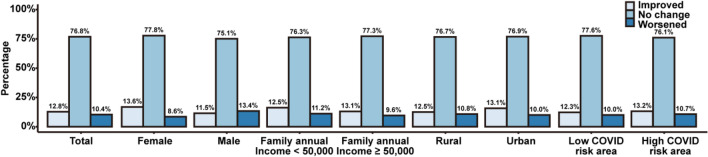
The change of severity of anxiety symptoms by sex, family income, urban/rural areas, and COVID-19 risk areas between 2019 and 2020. Notes: Statistically significant difference was found in total (*p*-value = 0.007), females (*p*-value < 0.001), individuals from annual income ≥ 50,000 families (*p*-value < 0.001), individuals from urban areas (*p*-value = 0.006), and individuals from high COVID-19 risk areas (*p*-value = 0.042).

Figure 2 shows the change of severity of anxiety symptoms by sex, family income, and COVID-19 risk areas between 2019 and 2020. For each around 77% of the participants’ anxiety symptoms stay unchanged, around 12% improved and 11% worsened. Males had higher proportions of individuals with worsen anxiety symptoms than those with improved ones, with 11.5% showing improved anxiety symptoms, 13.4% with worsen symptoms.

**Figure 3:**
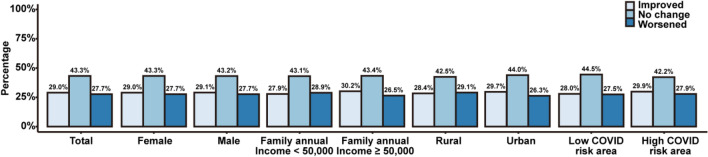
The change of severity of stress symptoms by sex, family income, urban/rural areas, and COVID-19 risk areas between 2019 and 2020. Notes: Statistically significant difference was found in individuals from annual income ≥ 50,000 RMB families (*p*-value = 0.008).

Figure 3 shows the change of severity of self-reported stress by sex, family income, and COVID-19 risk areas between 2019 and 2020. For each around 43% of the participants’ self-reported stress remain unchanged, 29% improved and 28% worsened. Higher proportions of individuals with worsen stress symptoms than those with improved symptoms were found in students from low-income families and those from rural areas.

**Figure 4:**
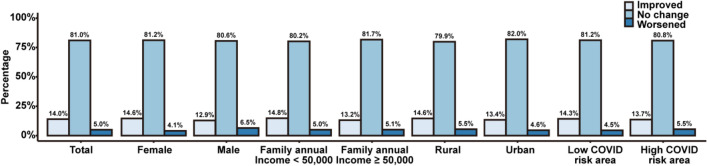
The change of sleep quality by sex, family income, urban/rural areas, and COVID-19 risk areas between 2019 and 2020. Notes: Statistically significant difference was found in total (*p*-value < 0.001), and all the subgroups (*p*-value < 0.001).

Figure 4 shows the change of sleep quality by sex, family income, and COVID-19 risk areas between 2019 and 2020. Around 81% of the participants’ sleep quality remain unchanged. There were statistically significant (*p* < 0.001) improvements of the undergraduates’ sleep quality in all the subgroups. Around 14% of the participants’ sleep quality improved. Around 5% of the participants’ sleep quality became worse. There was significant difference in the changing magnitude of sleeping quality in all the subgroups (*p* < 0.001).

**Figure 5:**
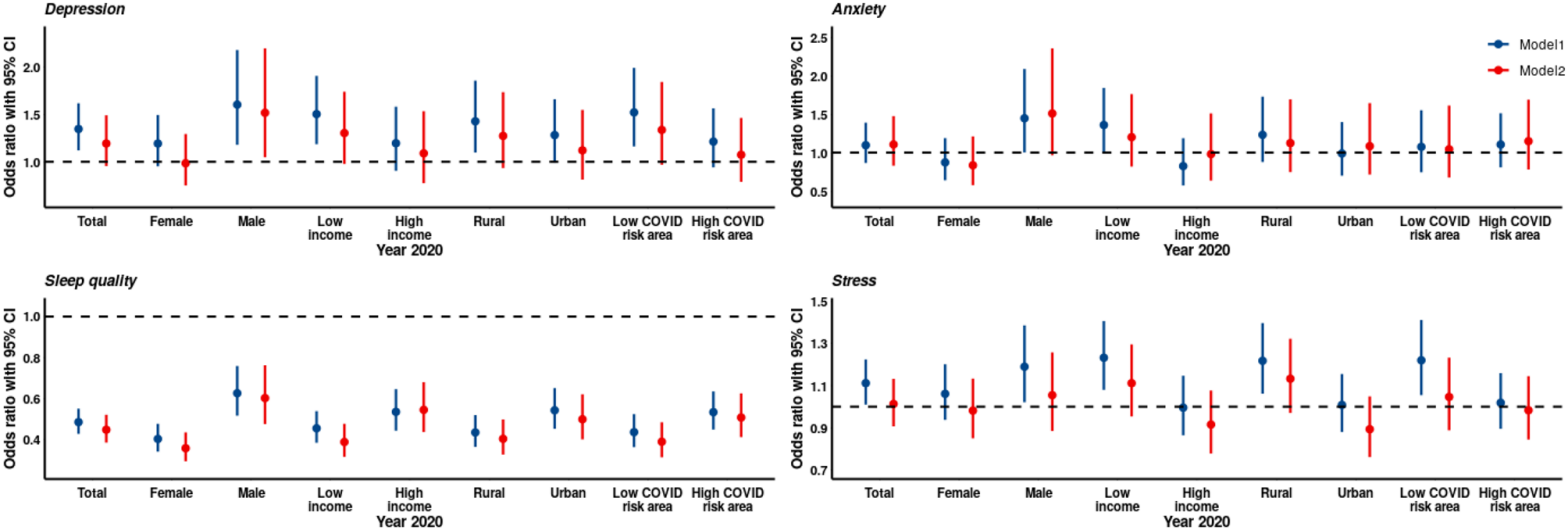
The association between survey year (2020 versus 2019) and moderate to severe depression, moderate to severe anxiety, stress, and good sleeping quality. Notes: Model 1 adjusted for age and sex. Model 2 adjusted for age, sex, exercise habit, time spent outdoors, and time spent on mobile gadgets.

Figure 5 shows the association between survey year (2020 versus 2019) and moderate to severe depression, moderate to severe anxiety, stress, and good sleeping quality.

Compared to year 2019, being in 2020 (model 1: adjusted for age and sex) was significantly associated with the improvement of sleeping quality in total [odds ratio (OR) 0.48, 95% confidence intervals (95% CI):0.43–0.55, *p* < 0.001] and in all the subgroups (*p* < 0.001). Year 2020 was significantly associated with severity of depression and self-reported stress in total, in males, in students from low income families, rural areas, and low COVID-19 risk areas. Year 2020 was significantly associated with severity of anxiety symptoms in males and in students from low income families. After adjusting for age, sex, exercise habit, time spent on mobile gadgets, and time spent outdoors, year 2020 still was significantly associated with the improvement of sleeping quality in total (OR:0.45, 95%CI:0.38–0.52, *p* < 0.001) and in all the subgroups (*p* < 0.001). Year 2020 was significantly associated with severity of depression symptoms in males (OR:1.52. 95%CI:1.05–2.20, *p* < 0.05). There was no significant difference in the ORs of the two models.

The association between year 2020 and mental health status using 5 points as cut-offs for anxiety and depression are shown in Figure S2. Year 2020 became protective factors for depression, anxiety symptoms and sleeping quality. When using 15 cases per million as the cut-offs for high/low pandemic areas, the results are shown in Figure S3-S4. Anxiety symptoms improved significantly in students from low COVID-19 risk areas (Figure S4).

## Discussion

This longitudinal cohort study investigated the mental health status, sleep quality and influencing factors before and after the COVID-19 mass quarantine. This study found no deterioration in mental health status among Chinese undergraduate students in 2020 after COVID-19 mass quarantine compared with the baseline measures in 2019. During the COVID-19 mass quarantine period, the length of staying outdoors significantly reduced. Time spent on mobile gadgets (i.e., mobile phones and tablets) increased considerably. The self-reported sleeping quality improved after the mass quarantine. Year 2020 was a protective factor for sleeping quality. Year 2020 was positively associated with the severity of depression symptoms in males.

The severity of anxiety symptoms was severe (1.0%), moderate (2.5%), and mild (17.1%) in our baseline survey. During the outbreak of COVID-19, a cross-sectional study among 7,143 college students of a medical school in China found the severity of anxiety was severe (0.9%), moderate (2.7%), mild (21.3%) respectively^19^. Consistently, previous studies indicated that the outbreak of infectious disease, such as severe acute respiratory syndrome (SARS) and middle East Respiratory Syndrome (MERS), was associated with negative psychological effects^20^. During the outbreak of infectious disease, infection fears, frustration, boredom, inadequate supplies could result in stress symptoms. A longitudinal study investigated the adverse mental health status, assessed by depression anxiety and stress scale (DASS-21), during the initial outbreak (31 January 2020 to 2 February 2020) and the peak of COVID-19 epidemic (28 February 2020 to 1 March 2020) among the general population in China and found no significant changes in the anxiety and depression scores^21^. Similarly, a cross-sectional survey of 2,330 primary school students in Hubei province found higher proportions of individuals having anxiety and depression symptoms than previous investigations found after restricting to home for 33.7 (2.1) days^22^. One or two months after the outbreak of COVID-19 when there were a lot of unknowns, studies showed deteriorations in the mental health status of various populations^21,22^. When it comes to the mandatory quarantine, the negative psychological effects can last several months to several years^23^. A study in Beijing investigated 549 hospital employees in 2006. The results found being quarantined during the outbreak of SARS was positively associated with increased odds of having a high level of depressive symptoms three years later^23^.

However, almost three months after the initial outbreak, the severity of depression and anxiety symptoms and self-reported stress showed no significant difference from the baseline survey results in this study. A study in Korea included 1,692 individuals who were isolated for two weeks during the MERS epidemics. Anxiety symptoms were evaluated with the GAD-7 scale during isolation and 4–6 months after isolation. The cut-off point was set as 10 points or higher. The result indicated that the isolated people appear to have recovered to normal levels of anxiety 4–6 months after removal from isolation^24^. Three or four months after the initial outbreak, the mental health status can recover from the distress reaction. The non-significant deterioration of mental health status in this study could possibly be due to sampling period. Another reason for this could possibly because the research subject in this study is undergraduate students. Undergraduate students generally have fewer responsibilities and economic burdens than independent adults. For example, a study compared the impact of event scale-revised (IES-R) score of 176 quarantined undergraduate students with 243 nonquarantined students during the mass infection of the 2009 H1N1 flu. There was no significant difference between the quarantined and nonquarantined group^18^. It could possibly because young adults have a high resilient ability to cope with unprecedented incidents.

Quarantine separates and restricts the movement of people who were exposed to a contagious disease to see if they become sick. The self-quarantine in this study was voluntary. During the mass quarantine, the time spent outdoors was less than that during the regular school period. Undergraduate students spent more time indoors. However, their exercise frequency did not show significant change. In addition, due to school closure, all the students were educated online at home, leading to prolonged phone occupying time. In short, during the mass quarantine, Undergraduate students spent more time at home playing or learning with their phones, and exercised regularly. The comfortable home environment, reduced academic stress, and survivor relief could explain the increased proportion of normal mental health status individuals. Survivor relief means someone feels relief and appreciation for their survival. In addition, individuals still can go outside which could effectively relief the mental distress.

However, the Household Pulse Survey in the United States of America used two-item Patient Health Questionnaire (PHQ-2) and two-item Generalized Anxiety Disorder (GAD-2) scale to collect information on mental health symptoms over the last 7 days between 14–19 May 2020. The results indicated that the anxiety and depression signs were more than tripled comparing with the baseline survey in January-June 2019 among 119,897 participants^25^. The different results could possibly be due to different study subjects, different disease control procedures or an increase in case count. For example, In January 2020 the United States had less than 10 COVID-19 cases. By the end of May 2020 there were more than 1.7 million cases (https://coronavirus.jhu.edu/map.html). Another study in the first quarantined community in the United States of America found that 48 h after the containment, 300 participants demonstrated elevated distress/anxiety levels. In addition, there was no difference in distress/anxiety levels between fully quarantine and partial quarantine individuals^26^. Another national-wide study in Australia also found very high prevalence of depression and anxiety symptoms-especially among people who had lost jobs or whose courses were cancelled among 13,829 adults from 3 April 2020 to 2 May 2020^27^.

In the subgroup results, there was no significant difference in depression symptoms in 2020 and in 2019. The depression symptoms significantly worsened in males. However, there were more females with improved depression symptoms than worsen depression symptoms. In addition, undergraduates from high income families showed more improved symptoms versus worsen symptoms than students from low-income families. Female and socioeconomic advantage undergraduates cope with the COVID-19 better than male and socioeconomic disadvantage undergraduates. One study has found gender differences in emotional responses to COVID-19. In this study, female participants had a tendency to self-report worries about the family whereas male participant often reported worries about economy^28^. The family well-being could be guaranteed during the mass quarantine in China. However, the financial situations were influenced. That could partly explain the improvements of females’ mental health status and the deterioration of male’s mental health status in this study. Consistently, a cohort study among employees of France’s National Electricity and Gas Company found that youths from low-income families had elevated rates of internalizing symptoms (including anxious/depressed syndrome, withdrawn behavior, and somatic complaints) at the follow-up (8 years after baseline) compared with baseline^29^. Later this cohort found lower family income was significantly associated with higher levels of depression in adolescents (13–21 years)^30^. Besides the present family poverty, family poverty over early life also predicts adolescent and young adult anxiety and depression^31^. In addition, our results found that there was no significant difference in the change of depression symptoms in students from different pandemic areas and from urban/rural areas.

The anxiety symptoms improved in 2020 than in 2019. The improvement was significant in females, in students from high income families, from urban areas, and from high COVID-19 risk areas. Similar as depression symptoms, females and socioeconomic advantage students cope with COVID-19 better. In addition, students from urban areas demonstrated significant improvement of anxiety symptoms. But there was no difference in the changing anxiety symptoms in urban/rural areas. This study did not find mental health discrepancy in undergraduates from urban or rural areas.

It is necessary to notify that students from high COVID-19 risk areas showed improved anxiety symptoms, that is probably due to the choice of cut-off point for high/low risk areas. In the sensitivity test, when we used 15 cases per million as cut-off point for high/low risk areas, anxiety symptoms were significantly improved in low risk areas.

The self-reported stress showed no significant difference between 2019 and 2020. However, in students from high income families the stress feelings significantly improved. Stress feelings deteriorated in students from low income families. The difference in urban/rural areas was significant, as the stress feelings for students from urban areas improved, but rural areas worsened. Individuals in high income families showed improvements in mental health. However, mental health deteriorates for those in low income families. COVID-19 affects the mental health of students from socioeconomic disadvantage families more severely. Consistently, Aaron et al. indicated that COVID-19 affect everyone unequally, and could exacerbate inequalities in the United States^32^.

The sleeping quality significantly improved in 2020 than in 2019. The improvement was consistent among different subgroups. A college dorm normally houses four to eight people in China. However, most students have private bedrooms at home. Since all the students were confined at home during the mass quarantine, the improved living condition could partly explain the improved sleeping quality. The improved sleeping quality could partly explain the non-significant change in mental health status scores, as Rosen indicated that sleeping quality was a behavior predictor of mental distress^26^.

The associations between year 2020 and mental health status and sleeping quality showed different results in two models. The consistent result is, independent of other factors, year 2020 could improve undergraduates’ sleeping quality. Previous cross-sectional studies found that quarantine due to 2019-nCoV pandemics in Italy was associated with sleep disorders^33^. Another cross-sectional survey among undergraduate students in Greece during 4–9 April 2020 indicated that 43% individuals reported deteriorate sleep quality^34^. Our cohort results disagree with these cross-sectional studies’ results. The different result is, without controlling for lifestyle characteristics (i.e., exercise habit, time spent outdoors, time spent on mobile gadgets), year 2020 was positively associated with severity of depression and stress in total and some subgroups, and severity of anxiety in males and in students from low income families. After controlling for lifestyle characteristics, year 2020 was only positively associated with severity of depression symptoms in males. The improvement of the mental health status in year 2020 could possibly be mediated by lifestyle changes.

Our study has several strengths. The prospective longitudinal design allows this study to give a repeatable result on the effect of COVID-19 on mental health. The longitudinal design is rare due to the unpredictability of the pandemic disease. Moreover, we used reliable statistic methods, and the generalized estimating equation is especially suitable for the repeated measurements. Thirdly, the results have significant realistic meanings. Our study demonstrates how COVID-19 quarantine directly impacted psychological health among undergraduate students.

There are limitations in this study. First, reporting bias-all the data were based on self-reported information. Poor mental health has been reported to contribute to academic dishonesty, substance abuse, and increased cynicism and decreased compassion among medical students^35^. Some undergraduate students may not want to report anxiety and depression in case it impacts upon their future^35^. Secondly, we did not consider academic pressure and employment pressure. As the participants were freshmen during the study period, the academic pressure and employment pressure were relatively low in comparison with higher grade. Thirdly, we used a non-validated instrument to measure stress so changes or non-changes may not be valid. The fourth, we arbitrarily used 6 cases per million population as COVID-19 high pandemic areas.

## Conclusion

No significant change in mental health status was observed among Chinese undergraduate students in 2020 after COVID-19 outbreak compared with the baseline measures in 2019, and there was an improvement in sleep quality and anxiety symptoms. This longitudinal study suggests that previous cross-sectional reports about adverse mental impacts of COVID-19 outbreak and mass quarantine might be exaggerated.

## Supplementary Information

Supplementary Information.
