# Supplementary material for: Mental health of new undergraduate students before and after COVID-19 in China
Source: Sci Rep. 2021 Sep 22;11:18783. doi: 10.1038/s41598-021-98140-3 (PMC8458482; doi:10.1038/s41598-021-98140-3)
Supplement: Supplementary file 1 — Supplementary Information. [file 41598_2021_98140_MOESM1_ESM.docx]

**Supplementary Materials**

**Mental health of new undergraduate students before and after COVID-19 in China**

Peng Lu Ph.D.^1^#, Lei Yang Ph.D ^2^#, Chongjian Wang Ph.D ^3^#, Guoxin Xia M.D.^4^, Hao Xiang Ph.D ^5^, Gongbo Chen Ph.D ^5^, Ning Jiang Ph.D ^6^, Tingting Ye Ph.D ^1^, Yucheng Pang Ph.D ^7^, Hongwei Sun Ph.D ^6^, Lailai Yan Ph.D^8^, Zhenguo Su M.D.^4^, Jane Heyworth Ph.D ^9^, Rachel Huxley Ph.D ^10^, Jane Fisher Ph.D ^1^, Shanshan Li Ph.D ^1^, Yuming Guo Ph.D^1^*

1.Department of Epidemiology and Preventive Medicine, School of Public Health and Preventive Medicine, Monash University, Melbourne, Victoria, Australia;

2. Department of Epidemiology and Statistics, School of Public Health, Hebei Medical University, Hebei Key Laboratory of Environment and Human health, Shijiazhuang, China;

3. School of Public Health, Zhengzhou University, Zhengzhou, Henan, China;

4. The Second School of Clinical Medicine, Binzhou Medical University, Yantai, Shandong, China;

5. Department of Global Health, School of Health Sciences, Wuhan University, 115 Donghu Road, Wuhan, Hubei, China;

6. School of Public Health and Management, Binzhou Medical University, Yantai, Shandong, China;

7.Department of Human Resources, Binzhou Medical University, Yantai, Shandong, China;

8.School of Public Health, Peking University, Beijing, China;

9. School of Population and Global Health, The University of Western Australia, Perth, Western Australia, Australia;

10.Deakin University, Melbourne, Victoria, Australia.

# These authors contributed equally to this paper.

^*^ Correspondence to: Yuming Guo: [Yuming.Guo@monash.edu](mailto:Yuming.Guo@monash.edu).


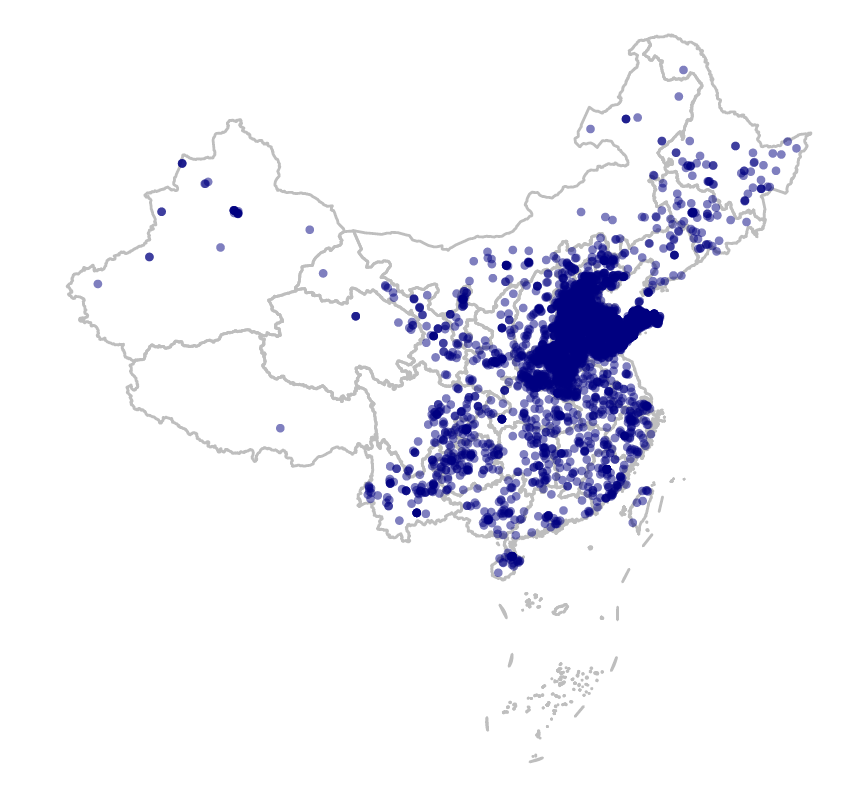


FigureS1: the location (home address) of the participants

Note: The base map was downloaded from Standard Map Service (SMS), hosted by China National Basic Geographic Information Centre (http://bzdt.ch.mnr.gov.cn/). The base map was open access.”

Table S1 The distribution of mental health status between 2019 and lockdown

| Mental health status | Anxiety |  |  |  | 0.007 |
| --- | --- | --- | --- | --- | --- |
|  |  | normal | 4,114 (79.4%) | 4,247 (82.0%) |  |
|  |  | mild | 887 (17.1%) | 744 (14.4%) |  |
|  |  | moderate | 130 (2.5%) | 140 (2.7%) |  |
|  |  | severe | 50 (1.0%) | 50 (1.0%) |  |
|  | Depression |  |  |  | 0.881 |
|  |  | normal | 3,648 (70.4%) | 3,758 (72.5%) |  |
|  |  | mild | 1,237 (23.9%) | 1,047(20.2%) |  |
|  |  | moderate | 200 (3.9%) | 240 (4.6%) |  |
|  |  | severe | 96 (1.9%) | 136 (2.6%) |  |
|  | Stress |  |  |  | 0.202 |
|  |  | No | 1,224(23.6%) | 1,538 (29.7%) |  |
|  |  | A little | 2,419(46.7%) | 1,982(38.3%) |  |
|  |  | mild | 1,068(20.6%) | 1,104(21.3%) |  |
|  |  | moderate | 392(7.6%) | 481(9.3%) |  |
|  |  | severe | 78（1.5%） | 72(1.5%) |  |


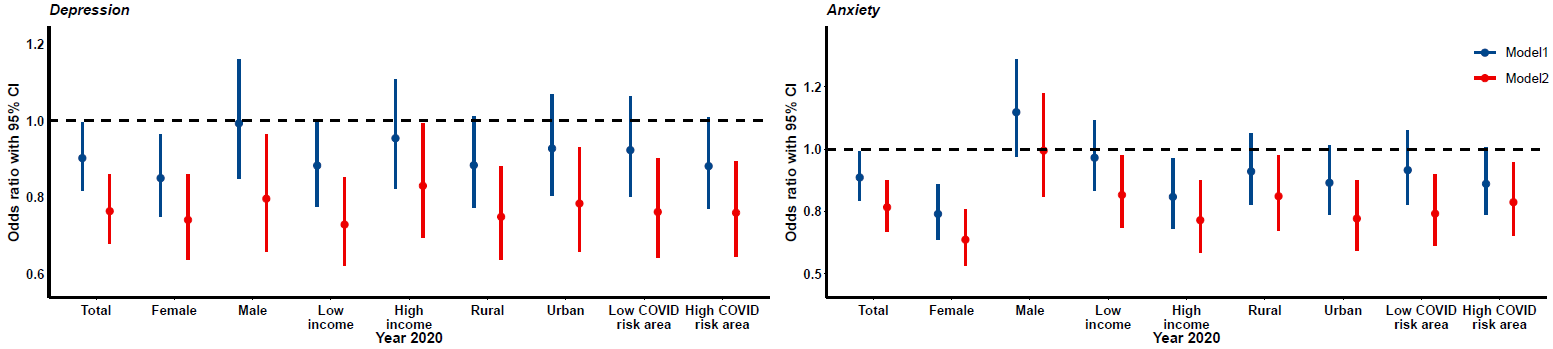


FigureS2: The association between year 2020 and mental health status using 5 points as cut-offs for anxiety and depression


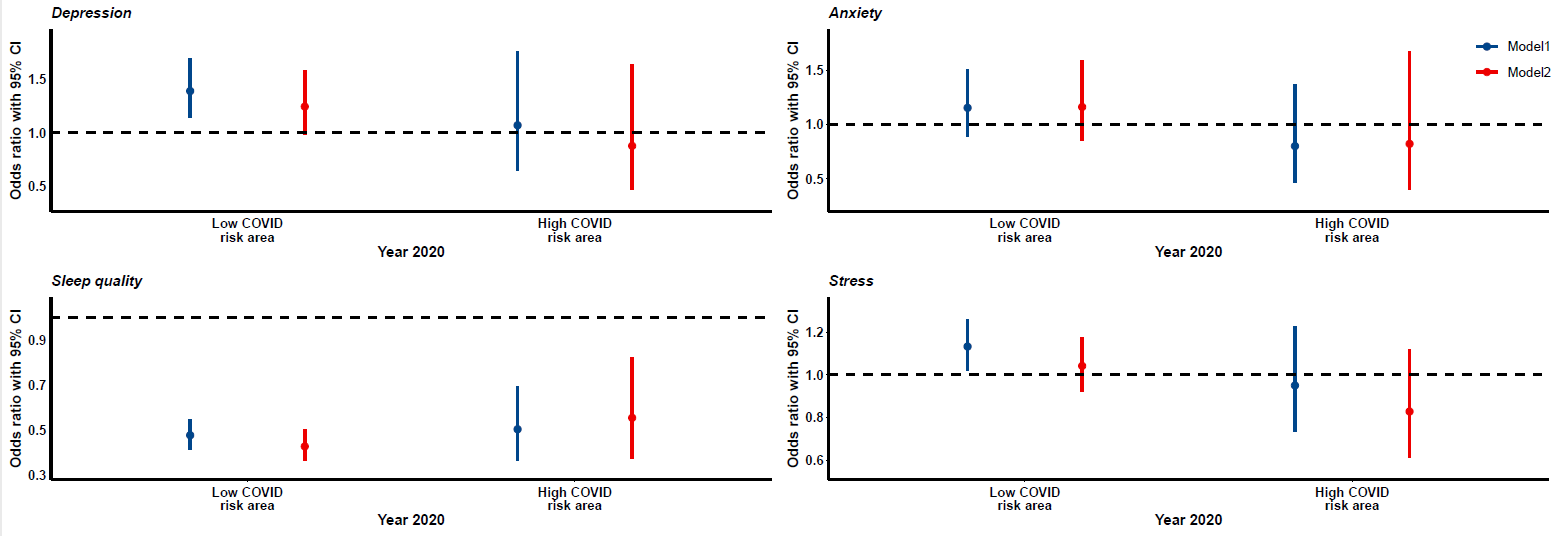


FigureS3: The association between year 2020 and mental health status using 15 cases per million as the cut-offs for high/low pandemic areas


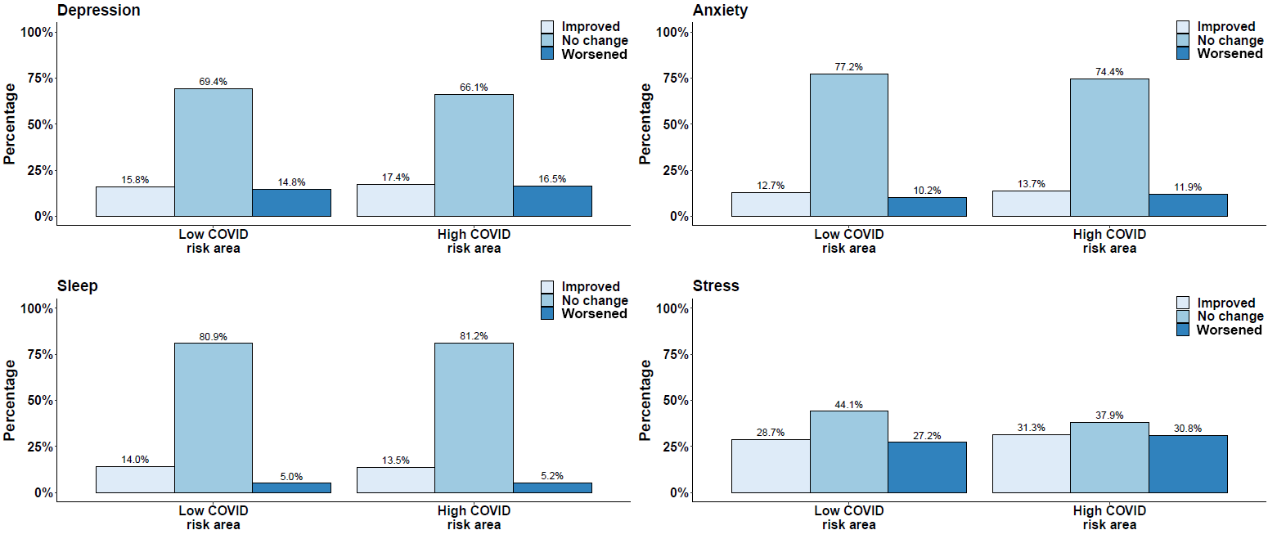


FigureS4: The change of depression, anxiety, stress, and good sleeping quality using 15 cases per million as the cut-offs for high/low pandemic areas.
